# Supplementary material for: Comprehensive profiling of the ligand binding landscapes of duplexed aptamer families reveals widespread induced fit
Source: Nat Commun. 2018 Jan 24;9:343. doi: 10.1038/s41467-017-02556-3 (PMC5783947; doi:10.1038/s41467-017-02556-3)
Supplement: Supplementary file 1 — Supplementary Information [file 41467_2017_2556_MOESM1_ESM.pdf]

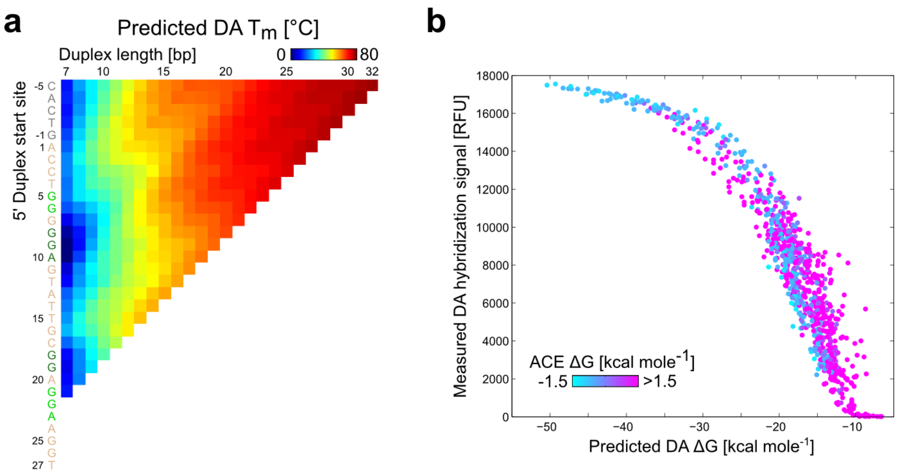

Supplementary Figure 1 | Correlation of ATP DNA DA hybridization signals to predicted DA hybridization and ACE self-hybridization free energies. (**a**) Heat map of predicted DA melting temperatures for perfect match ACEs, obtained using a nearest-neighbor method,[2] assuming 600 mM Na$^+$ and 1 μM total strand concentrations. (**b**) Scatter plot of measured DA hybridization signals as a function of predicted DA hybridization free energies (DA ΔG) and ACE self-hybridization free energies (ACE ΔG), for all perfect match and 12- and 15-mer single mismatched ACEs. Each DA is colored according to the DINAMelt-predicted[3] ACE self-hybridization duplex free energy (denoted ACE ΔG, from blue to magenta), which reflects the ability of the ACE to self-hybridize on the microarray surface and thereby interfere with aptamer hybridization. Free energies for ATP DNA DA aptamer-ACE duplexes and for ACE self-hybridized structures are reported for the most stable secondary structure predicted by DINAMelt using hybridization conditions of 25˚C, 300 mM Na$^+$ and 5 mM Mg^{2+}, and the free energy of the DINAMelt-predicted secondary structure is calculated relative to the free energy of the single-stranded state of the ACE or DA modeled. ACEs with more stable self-complementary secondary structures (negative ΔG values in blue) are expected to exhibit lower aptamer hybridization affinities than ACEs with unstable or no predicted secondary structures (single stranded ACEs with positive ΔG values in magenta) owing to an increase in expected duplex off rates.[4] Overall, the ATP DNA DA dataset closely follows a Langmuir isotherm,[5,6] suggesting that observed differences in ATP DNA DA hybridization signals were primarily driven by differences in ACE-aptamer duplex free energies, and not by aptamer secondary structure or ACE secondary structure. However, ACEs predicted to exhibit moderate self-complementarity (ΔG values < -1.0 kcal mole^{-1}, in blue) formed DAs that generally displayed slightly lower hybridization signals than ACEs without predicted self-hybridizing secondary structures (in magenta), as expected.

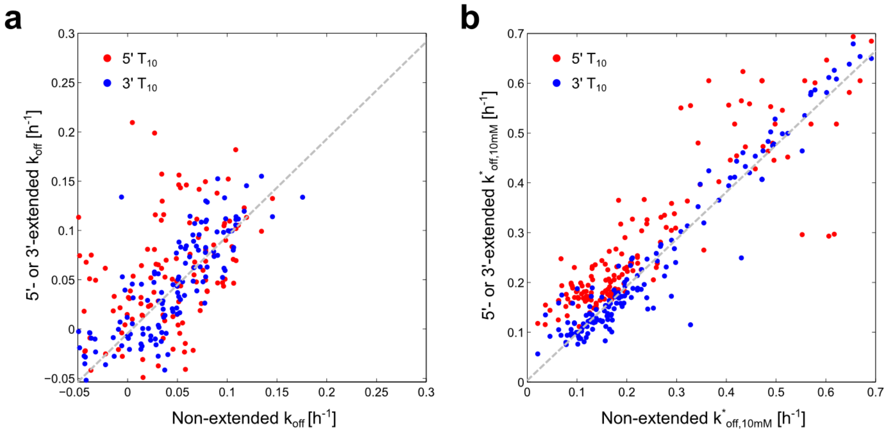

Supplementary Figure 2 | Effect of 5’ and 3’ T_{10} ACE extensions on ATP DNA DA ligand binding. (**a**) ATP DNA DA k_{off} rates for 5’ and 3’ T_{10}-extended ACEs as compared to non-extended ACEs. Dotted gray line represents a linear fit of the 3’ T_{10} extension dataset to the non-extended ACE dataset. A slight overall increase in k_{off} was observed for 5’ T_{10}-extended ACEs. (**b**) ATP DNA DA $k^{*}_{off,10mM}$ rates for 5’ and 3’ T_{10}-extended ACEs as compared to non-extended ACEs. Dotted gray line is a linear fit of the 3’ T_{10} extension dataset to the non-extended ACE dataset. 5’ T_{10} extended ACEs generated DAs with a slightly overall increased induced fit propensity under 10mM ATP. These findings suggest that increased intramolecular steric crowding of DAs, achieved by implementing 5’ T_{10}-extended ACEs, generally promotes DA duplex dissociation. 3’ T_{10}-extensions (resulting in DAs spaced a total of 35 T residues from the microarray surface) did not impact DA dissociation rates, as expected.

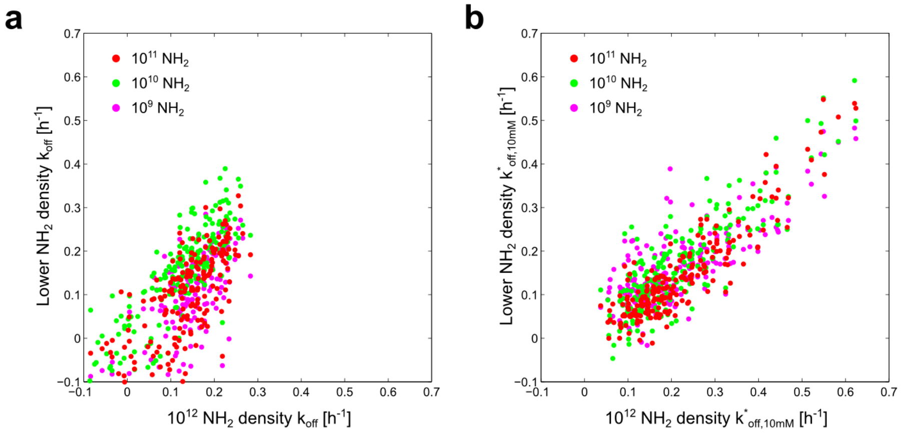

Supplementary Figure 3 | Effect of ACE surface density on ATP DNA DA ligand binding. (**a**) Comparison of DA k_{off} rates for microarrays synthesized on slides with surface densities of 10^{12} to 10^{9} active amines mm^{-2}. DAs on 10^{9} arrays generally displayed slightly lower rates of duplex dissociation under buffer-only conditions. (**b**) Comparison of DA $k^*_{off,10mM}$ rates for microarrays synthesized on slides with surface densities of 10^{12} to 10^{9} active amines mm^{-2}. DAs across slide surface densities displayed similar ligand-induced dissociation rates under 10 mM ATP. These results suggest that ACE surface density minimally impacts microarray-based DA ligand binding kinetics.

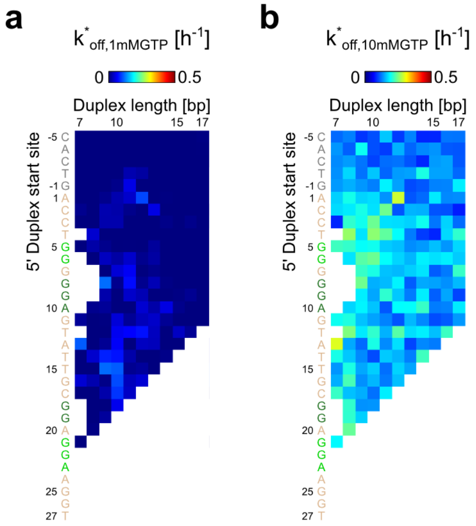

Supplementary Figure 4 | ATP DNA DA induced fit landscape obtained using GTP as a low-affinity control purine ligand. In contrast with the rich induced fit landscape observed for ATP DNA DAs subject to ATP (**Fig. 2d**), (**a**) incubation with 1 mM GTP did not increase DA duplex dissociation rates, and (**b**) 10 mM GTP led to only small increases (generally $k^*_{off,10mMGTP} < 0.15\ h^{-1}$) in $k^*_{off,10mMGTP}$ for ACEs hybridized to site II of the aptamer. Overall, the GTP-dependent induced fit landscape was much weaker than observed for ATP, as expected given the much lower affinity of the ATP DNA aptamer for GTP than ATP, and in-line with a previously observed 4- to 5-fold difference in signaling for a solution-based ATP DNA DA when subject to 10 mM ATP vs. 10 mM GTP.[1]

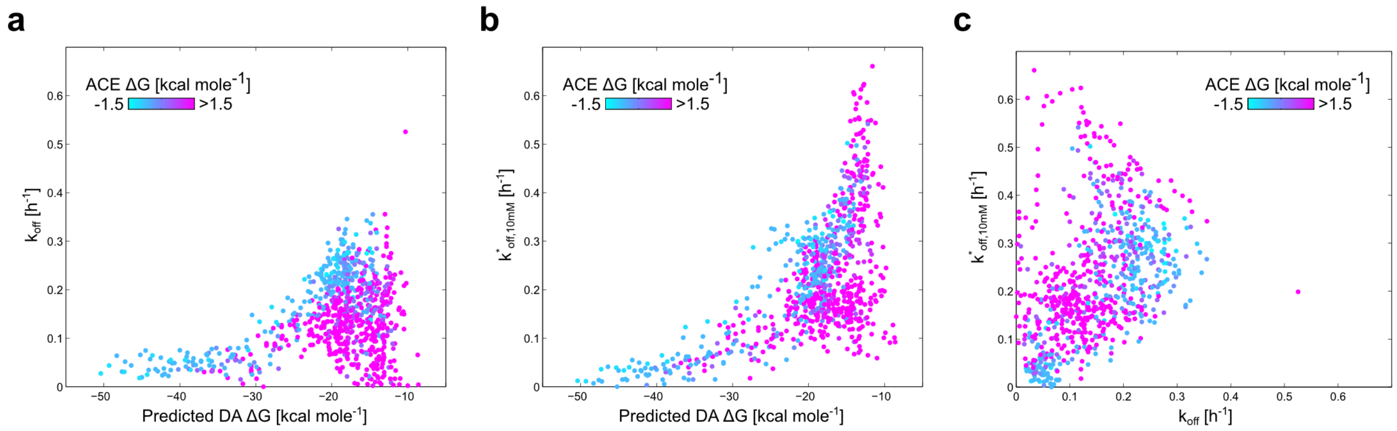

Supplementary Figure 5 | Correlation of ATP DNA DA k_{off} and k^*_{off} landscapes to predicted DA hybridization and ACE self-hybridization free energies. (**a**) DA duplex dissociation rates under buffer-only conditions (k_{off} rates) plotted as a function of DA hybridization free energies (DA ΔG), with each data point colored by ACE ΔG as in **Supplementary Fig. 1b**. Generally, k_{off} correlated to DA hybridization free energies, with shorter ACEs experiencing higher k_{off} rates. Additionally, ACEs more prone to self-hybridization (blue) generated DAs with increased k_{off}. (**b**) DA dissociation rates under saturating ATP conditions ($k^*_{off,10mM}$) plotted as a function of DA hybridization free energies, with each data point colored by ACE ΔG as in **Supplementary Fig. 1b**. Shorter ACEs generally yielded higher $k^*_{off,10mM}$ rates, however this behavior was highly ACE-specific. Interestingly, ACEs predicted to self-hybridize into stable secondary structures (blue) did not include a number of short DAs exhibiting the highest $k^*_{off,10mM}$ rates that are predicted to have no self-hybridizing secondary structure (in magenta). (**c**) $k^*_{off,10mM}$ plotted as a function of k_{off} for ATP DNA DAs, with each data point colored by ACE ΔG as in **Supplementary Fig. 1b**. A moderate correlation of $k^*_{off,10mM}$ to k_{off} is observed that is independent of ACE ΔG, however a small group of ACEs regulating the highest level of induced fit (and with low k_{off}) appear as outliers to this trend.

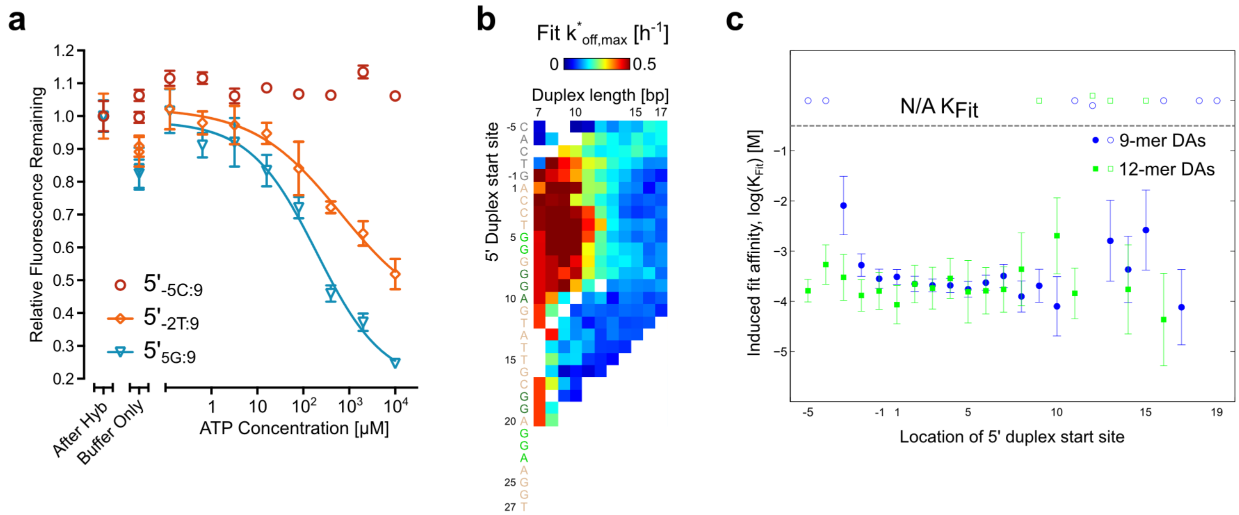

Supplementary Figure 6 | Quantitative induced fit ligand binding landscape of ATP DNA DAs obtained using a serial dilution of ATP over two ACE-Scan microarrays. (**a**) An 8-point serial dilution curve (10 mM to 0.124 μM ATP) obtained for three 9-mer ACEs with differing degrees hybridization to the aptamer binding site II. Lines represent curve fits to DA-specific datasets; error bars represent 1 standard error obtained from the 5 or 6 replicate DAs at each experimental condition. As previously observed, the $5'_{-5C:9}$ DA (red circles) exhibited no induced fit binding, whereas the $5'_{-2T:9}$ DA (orange diamonds), which hybridizes to guanosine bases present in binding site II of the aptamer, exhibited a high degree of induced fit.[7] The site II-hybridizing $5'_{5G:9}$ DA (blue triangles), which hybridizes throughout the dual ATP binding pocket, exhibited the maximum $k^*_{off,10mM}$ rate amongst 9-mer ATP DNA DAs. (**b**) Comprehensive $k^*_{off,max}$ 5’ heat map for 7- to 17-mer ACEs generated by fitting datasets from 8 ATP dilutions obtained using two ACE-Scan microarrays. White tiles represent DAs removed from the data analysis due to not passing quality control (Methods). As expected, the $k^*_{off,max}$ landscape obtained was similar to the $k^*_{off,10mM}$ landscape (**Fig. 2d**). (**c**) Comparison of experimentally measured K_{Fit} values for all 9-mer (blue circles) and 12-mer (green squares) perfect match ACEs. DAs for which K_{Fit} could not be reliably fit by the two-parameter non-linear regression were removed from the dataset and are shown above the dashed gray line as empty circles. K_{Fit} error bars represent 95% confidence intervals (+/- 2 standard deviations) obtained from the non-linear regression (see Methods and Data Availability). Higher uncertainties in K_{Fit} were obtained for DAs with lower $k^*_{off,max}$ values. K_{Fit} values in the 100-300 μM range observed for DAs promoting induced fit are in agreement with values previously obtained for $5'_{-2T:9}$ and $5'_{-5C:12}$ DAs using in-house fabricated microarrays[7].

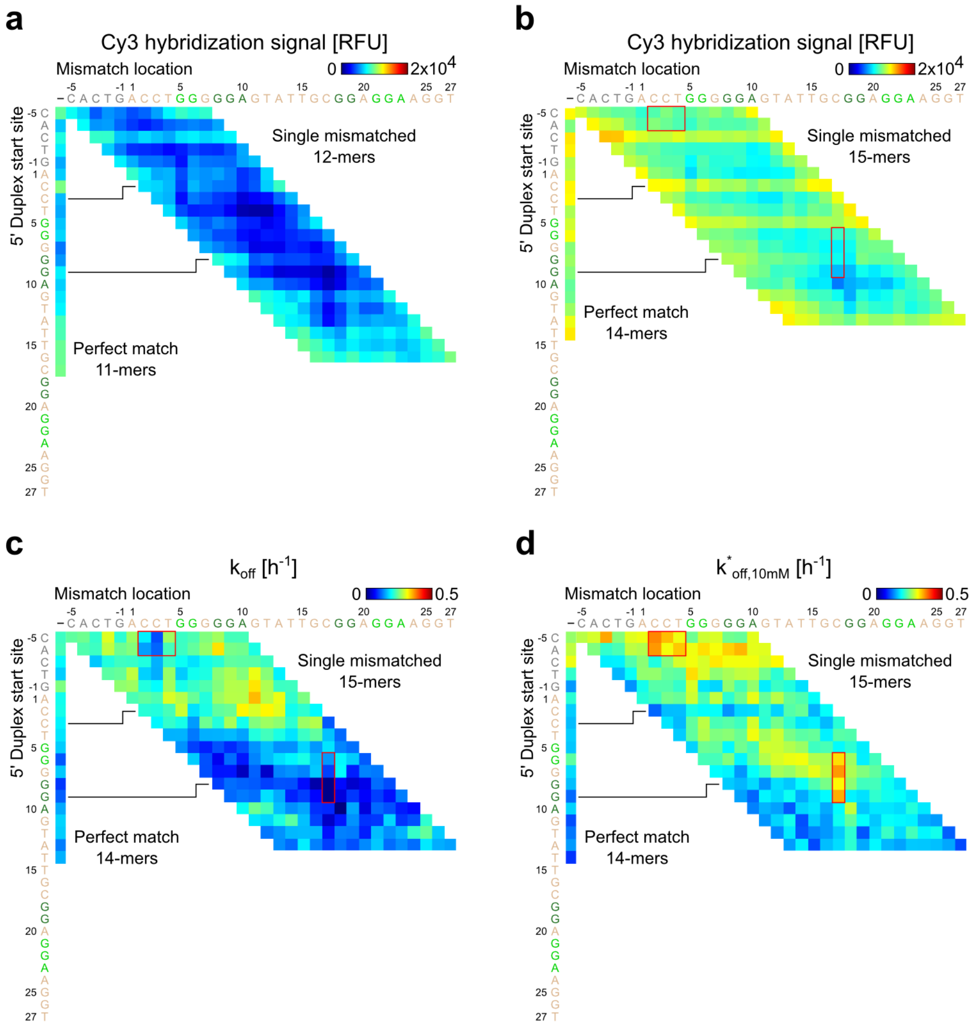

Supplementary Figure 7 | Hybridization affinity and ligand binding landscapes of ATP DNA DAs engineered with single mismatched ACEs. (**a**,**b**) Hybridization signal of (**a**) 12-mer and (**b**) 15-mer single mismatched ATP DNA DAs. Single-mismatched ACEs yielded lower hybridization affinities than perfect match ACEs (**Fig. 2b**), however 12- or 15-mer ACEs with mismatches near the 5' or 3' end of the ACE generated similar hybridization signals to 11- or 14-mer perfect match ACEs, respectively (leftmost column in **a**,**b** heatmaps). (**c**) 15-mer single mismatched ATP DNA DA dissociation rates under buffer-only conditions (k_{off} rates). (**d**) Increase in DA duplex dissociation rates under buffer + 10 mM ATP incubation ($k^{*}_{off,10mM}$ rates). The 15-mer single mismatched ACEs promoting the highest level of induced fit are hybridized at the 5' extreme of the ATP DNA aptamer sequence, whereas a set of 15-mer mismatched ACEs hybridized to binding site II displayed moderate induced fit propensity. These findings are in good agreement with the $k^{*}_{off,10mM}$ ligand binding landscapes obtained for perfect match 14-mer (leftmost column) and 15-mer ACEs (**Fig. 2d**). Using these landscapes, it is also possible to identify DAs expected to exhibit excellent sensitivity based on high hybridization affinities, low k_{off}, and high induced fit (e.g. 5'$_{-5C--4A:15:2C-4T}$ and 5'$_{6G-9G:15:17C}$ DAs in red boxes in **b**,**c**,**d**)

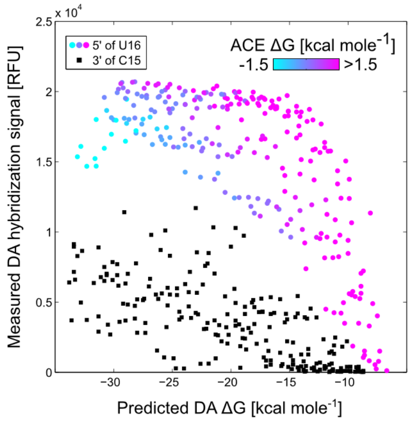

Supplementary Figure 8 | Hybridization affinity of ATP RNA DAs plotted as a function of predicted hybridization free energy, ACE location, and ACE self-complementarity. ACEs with 5’ duplex start sites 5’ before (i.e. 5- of) U16 (**see Fig. 4**) are shown as colored circles as a function of ACE ΔG, as in **Supplementary Fig. 1b**, whereas ACEs with 5’ duplex start sites after C15 are shown as black squares. ACEs with 5’ duplex start sites after C15 (black squares) displayed a clearly inhibited hybridization affinity, reflecting the more stable intrastrand secondary structure of the stem-loop region of the ATP RNA aptamer hybridized by these ACEs. ACEs with 5’ duplex start sites before U16 (circles) followed a Langmuir isotherm, however a clear influence of ACE self-complementarity was observed, with ACEs predicted to self-hybridize (ACE ΔG < 0) exhibiting lower hybridization signals.

a **b**

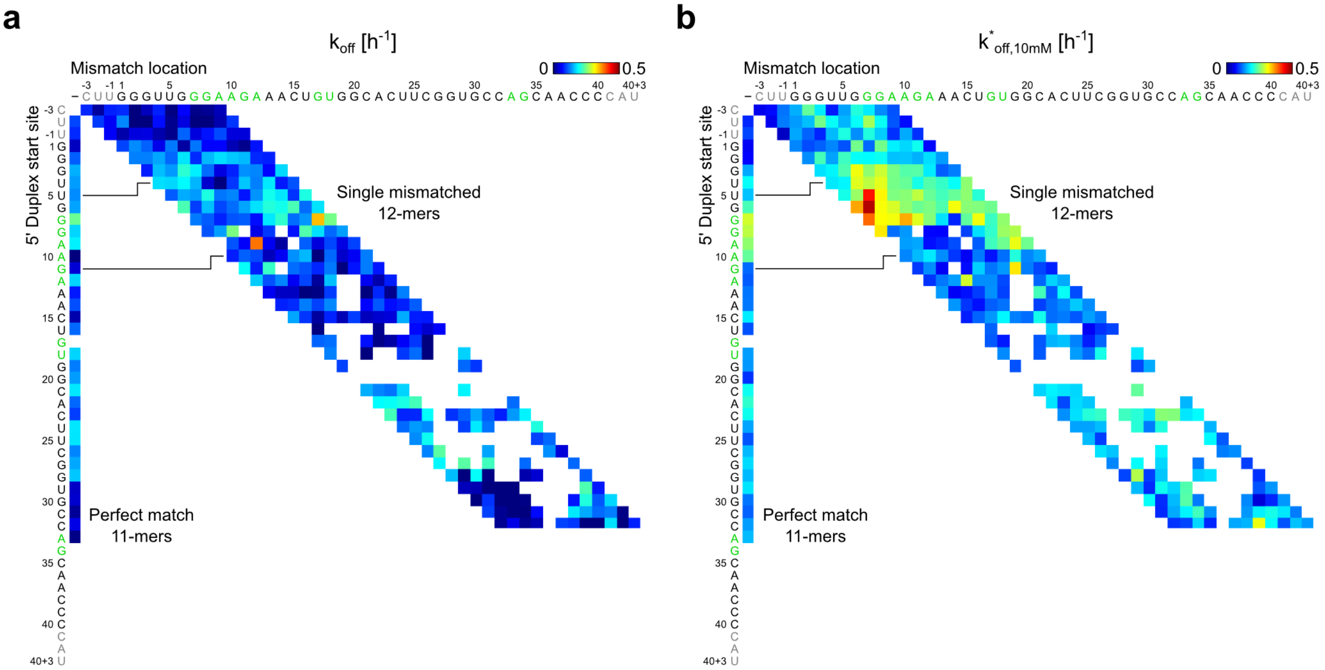

Supplementary Figure 9 | Ligand binding landscapes of ATP RNA DAs engineered with 12-mer single mismatched ACEs. Heat maps showing (**a**) DA dissociation rates under buffer-only conditions (k_{off}) and (**b**) increase in DA dissociation rates under 10 mM ATP ($k^*_{off,10mM}$). White tiles represent DAs that did not pass quality control and were removed from the analysis (Methods). The k_{off} and $k^*_{off,10mM}$ profiles reveal that single base-mismatched 12-mer ACEs (particularly ACEs that start 5' duplexing just 5' of the ATP RNA aptamer ligand binding pocket) increase the induced fit propensity of ATP RNA DAs, while maintaining low DA dissociation rates under buffer-only conditions.

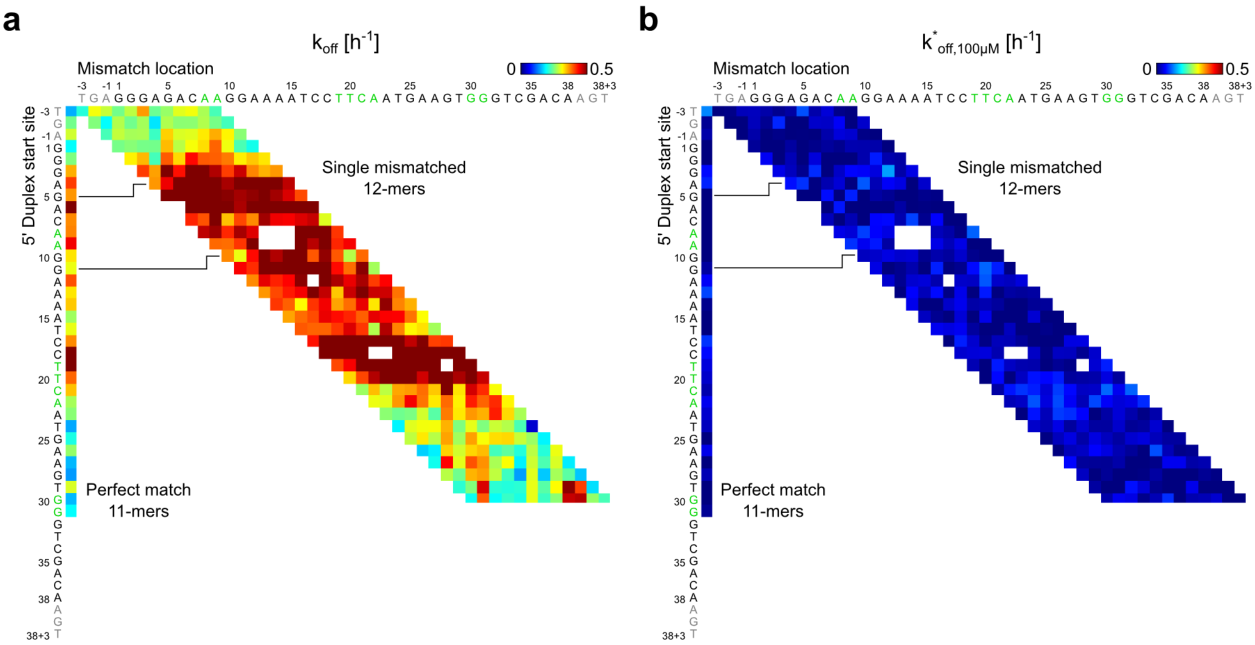

Supplementary Figure 10 | Ligand binding landscapes of cocaine RNA DAs engineered with 12-mer single mismatched ACEs. Heat maps showing (**a**) the DA dissociation rates of single mismatched 12-mer DAs under buffer-only conditions (k_{off} landscape), and (**b**) increase in dissociation rates for single mismatched 12-mer DAs subject to 100 µM cocaine ($k^{*}_{off,100\mu M}$ landscape). White tiles represent low quality DAs that did not pass quality control and that were removed from the analysis (Methods). The introduction of single mismatches into ACEs for cocaine DNA DAs led to high DA dissociation rates under buffer-only conditions (a further increase as compared to perfect match 11-mers), however mismatched ACEs did not promote any induced fit ligand binding in cocaine DNA DAs.

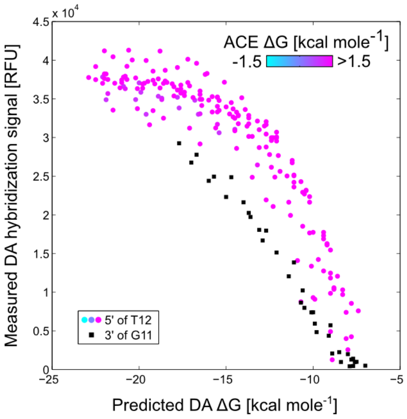

Supplementary Figure 11 | Correlation of TBA DA hybridization signals with predicted duplex free energies, ACE location, and ACE self-complementarity. ACEs with 5' duplex start sites before T12 are shown as circles colored as a function of ACE ΔG (as in **Supplementary Fig. 1b**), whereas ACEs with 5' duplex start sites after G11 are shown as black squares. The lower hybridization affinity of ACEs with 5' duplex start sites after G11 (black squares) may be due to increased steric crowding of hybridized TBA, which is predicted to have a pre-formed G-quadruplex that is directed towards the microarray surface when hybridized to these ACEs. TBA ACEs were not predicted to self-hybridize (> 0.5 kcal $mole^{-1}$ ACE ΔG values predicted for all 7- to 15-mer ACEs studied here).

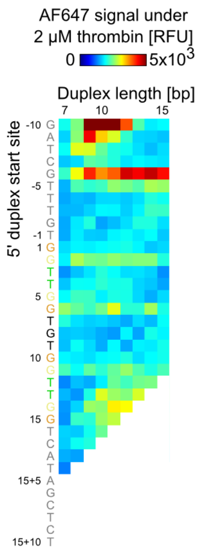

Supplementary Figure 12 | Binding capacity of 7- to 15-mer TBA DAs for AF647-labeled thrombin. The absolute signal of bound thrombin molecules per ACE was acquired for DAs after incubation of 2 µM labeled thrombin for 1 h.

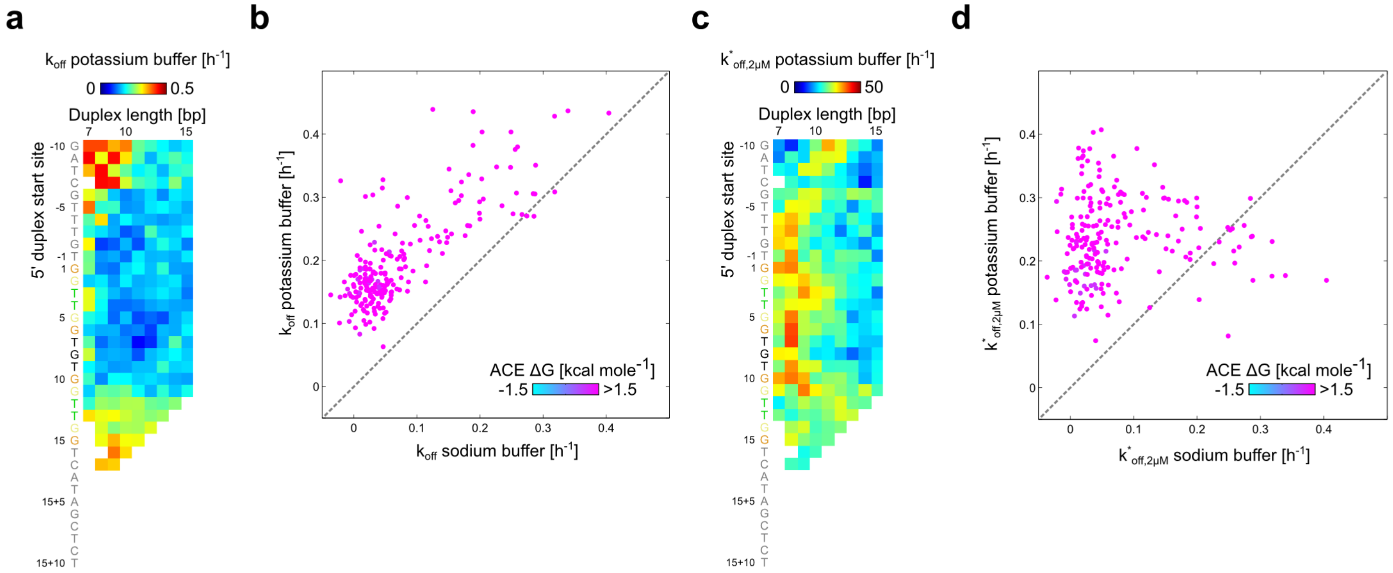

Supplementary Figure 13 | Cation-dependence of TBA DA binding landscapes. (**a**) Heat map of DA duplex dissociation rates under potassium buffer-only conditions. (**b**) Comparison of potassium vs. sodium cation on buffer-only DA k_{off} rates. Data points are colored by predicted ACE ΔG as in **Supplementary Fig. 1b**, and a 1:1 trend line is included for reference (dashed gray line). Compared to sodium, potassium is known to stabilize ligand-free TBA and ligand-bound thrombin-TBA structures, while also slightly perturbing the interfacing of TBA with thrombin exosite I[8]. Here, potassium buffer leads to higher k_{off} rates for TBA DAs. (**c**) Induced fit landscape of TBA DAs subject to 2 μM thrombin in potassium buffer. Including potassium as a cation instead of sodium leads to a broader induced fit landscape for TBA DAs, with up to 14-mer ACEs generating DAs that exhibit moderate $k^*_{off,2\mu M}$ rates. (**d**) Comparison of potassium vs. sodium as a buffer cation on TBA DA $k^*_{off,2\mu M}$ rates. Data points are colored by predicted ACE ΔG as in **Supplementary Fig. 1b**, and a 1:1 trend line is included for reference (dashed gray line). Generally, potassium as a cation, as compared to sodium, promoted an increased and broader induced fit profile in TBA DAs.

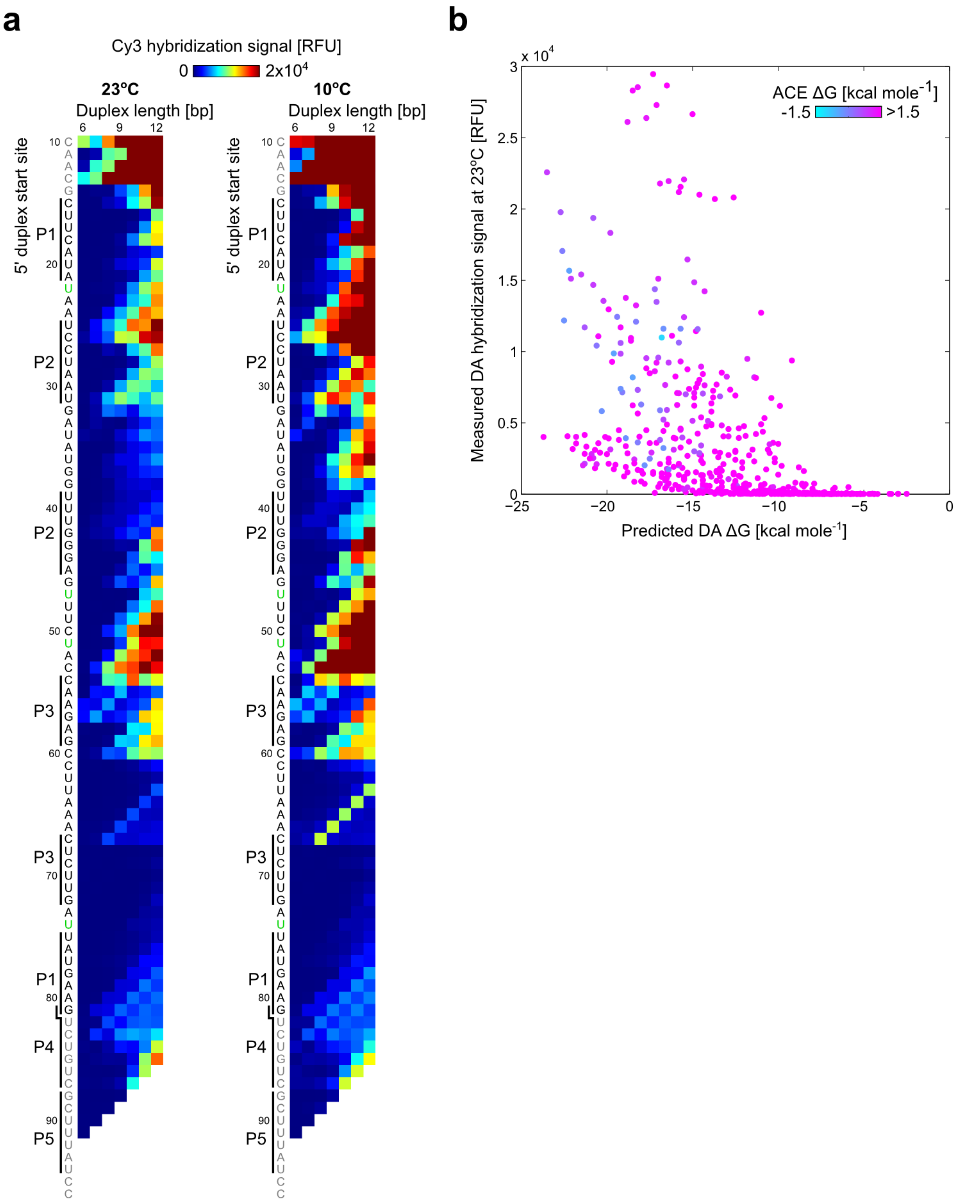

Supplementary Figure 14 | Hybridization affinity landscapes of *add* DAs. (**a**) Heat maps of the hybridization signal of *add* DAs obtained at 23˚C and 10˚C in 4xSSC buffer (Methods). As expected, a clear influence of aptamer secondary structure and temperature is observed, with double-stranded regions of the aptamer exhibiting lower hybridization affinities, while higher fluorescence signals were observed for a lower hybridization temperature. Bases that promote ACE hybridization nucleation are visible as horizontal or diagonal features. (**b**) Correlation of *add* DA hybridization affinities at 23˚C with DINAMelt-predicted duplex free energies. Data points are colored by predicted ACE ΔG, as in **Supplementary Fig. 1b**. While many short ACEs tested were found to hybridize poorly to the *add* riboswitch aptamer, a number of ACEs predicted to form stable duplexes with the aptamer (predicted DA ΔG < -10 kcal mole^{-1}) exhibited correspondingly high hybridization signals, identifying these as suitable ACEs for the generation of stable DAs.

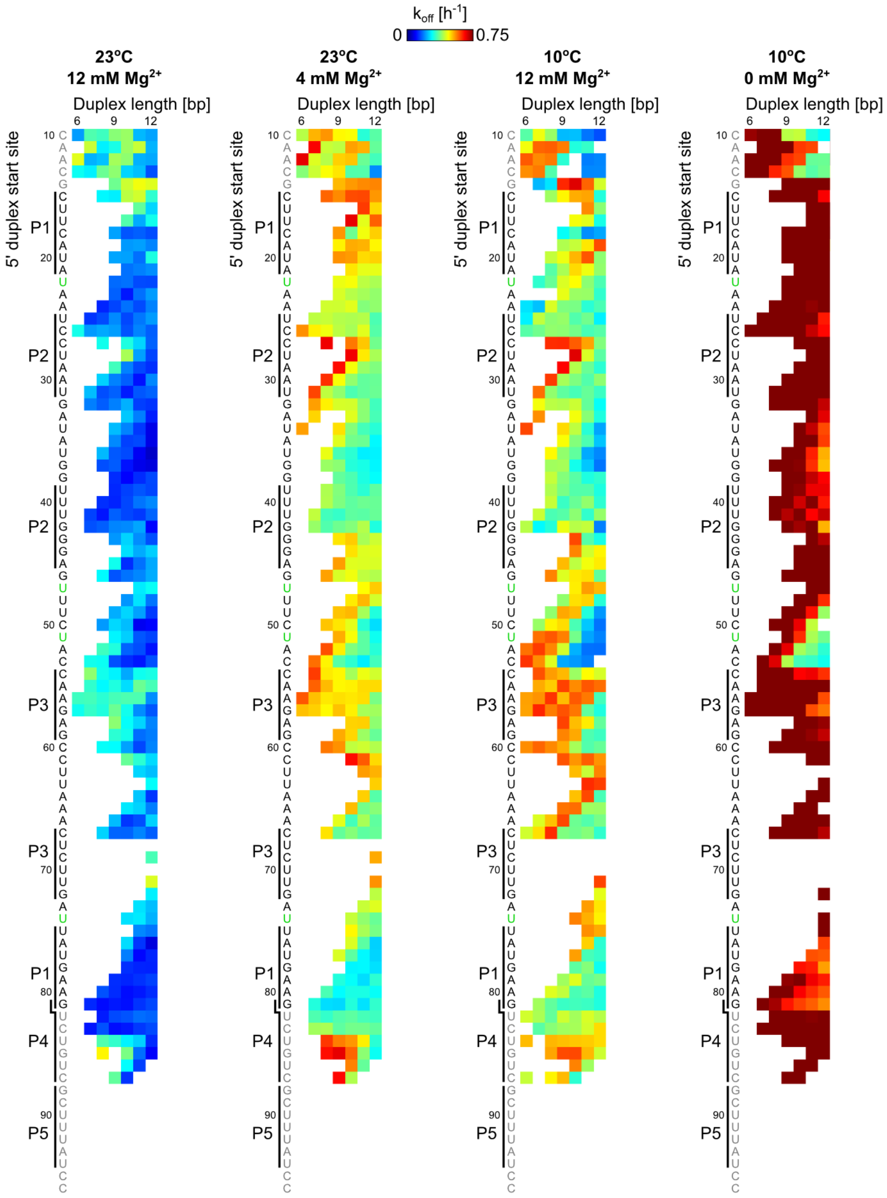

Supplementary Figure 15 | Duplex dissociation landscapes of *add* DAs under buffer-only conditions for a range of magnesium concentrations and temperatures. The k_{off} landscapes are similar in appearance, but highly dissimilar in magnitude across the different buffer-only conditions tested here, with decreasing magnesium concentration and decreasing temperature increasing *add* DA k_{off} rates.

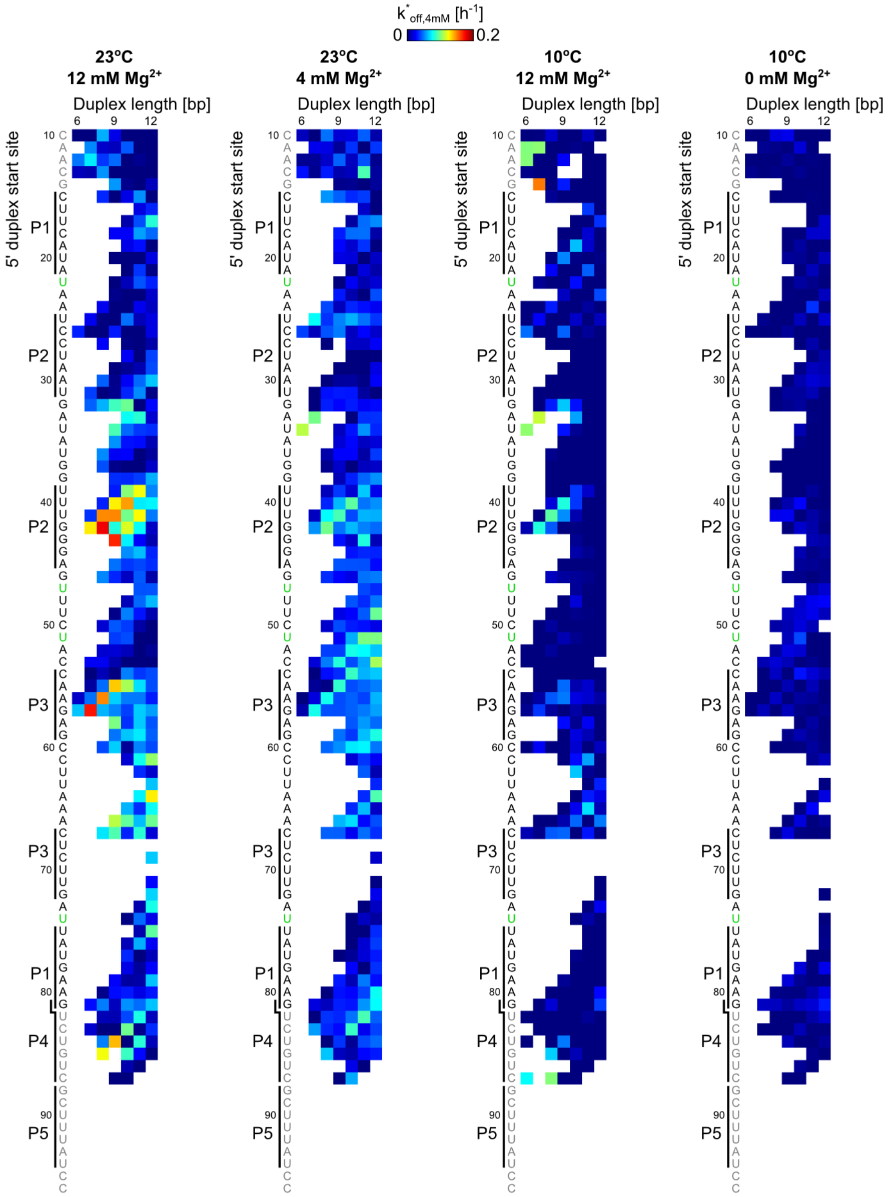

Supplementary Figure 16 | Duplex dissociation landscapes of *add* DAs under 4 mM adenine conditions for a range of magnesium concentrations and temperatures. The $k^*_{off,4mM}$ landscape was found to be highly dependent on temperature and Mg^{2+} concentration, with increasing magnesium concentration and increasing temperature promoting induced fit for a small number of *add* DAs.

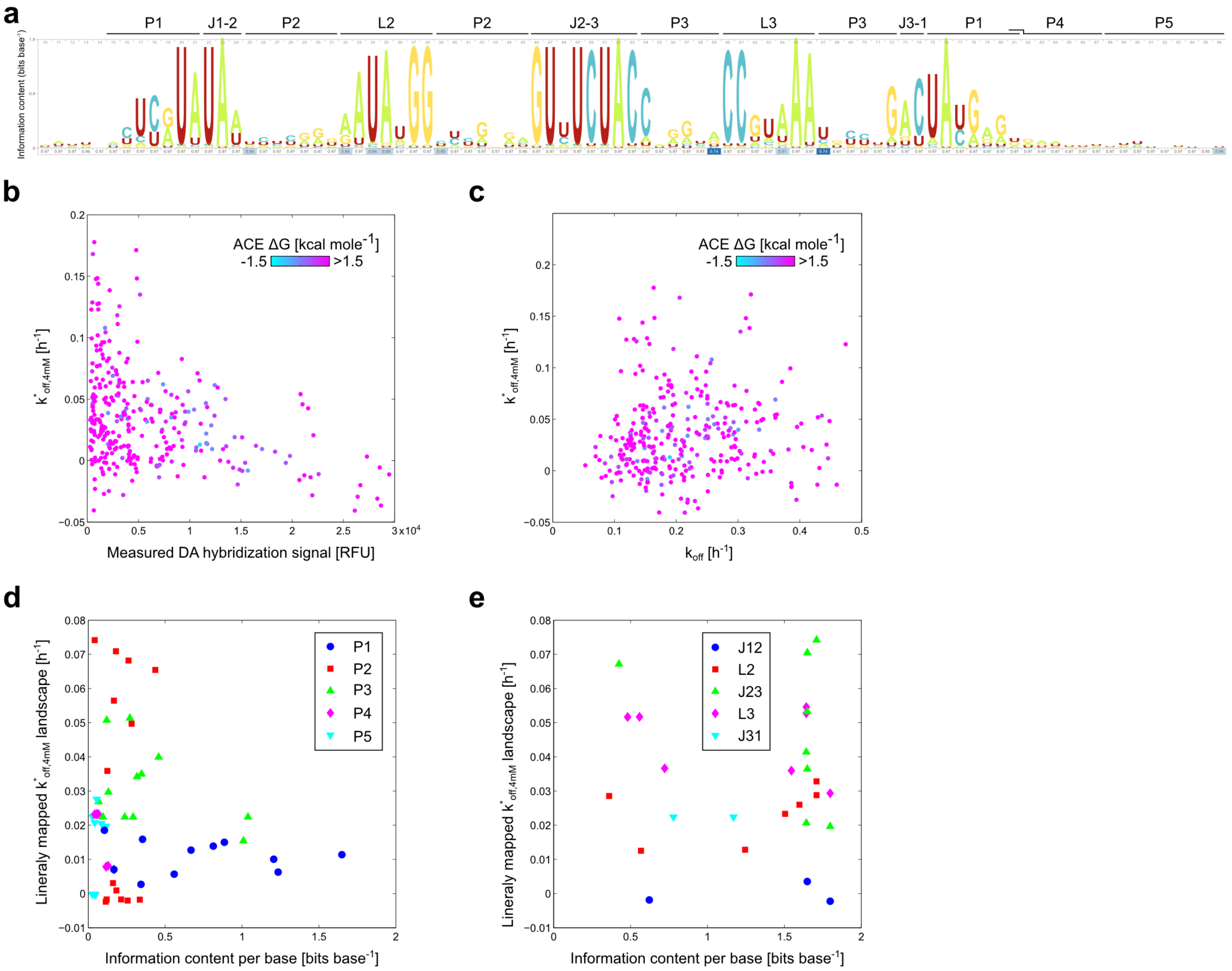

Supplementary Figure 17 | Correlation of the *add* DA induced fit ligand binding landscape to aptamer structure and sequence conservation scores. (**a**) Sequence logo of the purine riboswitch family, indicating sequence conservation as the information content per nucleotide per position (bits/base) and conserved position occupancy. The logo was created using Skylign, based on the seed sequences available in the Rfam database (RF00167) (Methods). (**b**,**c**) Comparison of *add* DA $k^{*}_{off,4mM}$ rates at 23˚C and 12 mM Mg^{2+} conditions to the (**b**) measured DA hybridization signal (23˚C, 4xSSC buffer) and (**c**) DA k_{off} rates (23˚C and 12 mM Mg^{2+}). Data points are colored by predicted ACE ΔG, as in **Supplementary Fig. 1b**. A weak inverse correlation was observed between $k^{*}_{off,10mM}$ and hybridization affinity, whereas a weak positive correlation between $k^{*}_{off,10mM}$ and k_{off} was observed. Overall, the *add* DA induced fit landscape was highly ACE-specific. (**d**,**e**) Forward linear mapping of 9-mer ACE induced fit landscapes (under 4 mM adenine, 23˚C and 12 mM Mg^{2+}) to the sequence conservation scores of purine riboswitches, shown for (**d**) double stranded and (**e**) single stranded regions of the *add* riboswitch aptamer (Methods). No correlation between induced fit and sequence conservation scores was observed for single or double stranded regions of the aptamer. Rather, ACEs hybridizing to specific aptamer regions (3' half of the P2 stem and bases in the J23 junction, the P3 stem, and the L3 loop) were found to promote the highest levels of induced fit ligand binding in *add* DAs. This is also depicted by the induced fit heat maps shown in **Supplementary Fig. 16**.

Supplementary Table 1: Complete sequences of aptamers used in this study

**Aptamer**	**Sequence*** **(5' to 3' IDT notation)**	**Purification**
ATP DNA**	/5Cy3/TCACTG<u>ACCTGGGGGAGTATTGCGGAGGAAGGT</u>	HPLC
ATP RNA***	/5Cy3/CUU<u>GGGUUGGGAAGAAACUGUGGCACUUCGGUGCCAGCAACCC</u> CAU	RNase-free HPLC
Cocaine DNA	/5Cy3/TGA<u>GGGAGACAAGGAAAATCCTTCAATGAAGTGGGTCGACA</u>AGT	HPLC
TBA	/5Cy3/GATCGTTTGT<u>GGTTGGTGTGGTTGG</u>TCATAGCTCT	HPLC
add riboswitch***	/5Cy3/CAACG<u>CUUCAUAUAAUCCUAAUGAUAUGGUUUGGGAGUUUCUA CCAAGAGCCUUAAACUCUUGAUUAUGAAG</u>UCUGUCGCUUUAUCC	RNase-free HPLC

* The consensus aptamer sequence is underlined.
** The ATP DNA aptamer sequence includes a 5' thymidine that was never duplexed in ACE-Scan experiments.
*** The aptamer was synthesized using the IDT ribonucleotide identifiers rA, rU, rG and rC.

Supplementary Note 1: Additional validation experiments for ATP DNA DAs

We investigated the effect of ACE immobilization on DAs by systematically scanning 8- to 15-mer ACEs with additional T_{10} extensions synthesized on the 5' end (10-base single-stranded extension) or 3' end (10-base additional linker length) of the ACE. A slight overall increase in duplex dissociation rates and induced fit (k_{off} and $k^{*}_{off,10mM}$) was observed for 5'-extended ACEs (**Supplementary Fig. 3**), suggesting that increased intramolecular steric crowding of DAs by 5' ACE extensions may promote duplex dissociation. We also tested the impact of ACE surface density using microarrays synthesized on slides with 10^{12} to 10^{9} amines mm^{-2}, corresponding to an average ACE spacing of ~1 to 100 nm (per microarray manufacturer), however a minimal impact of ACE surface density was observed (**Supplementary Fig. 4**). Of note, DAs on microarrays with surface densities of less than 10^{10} amines mm^{-2} exhibited very low hybridization signal intensities, which led to high uncertainties in fluorescence signal changes, limiting the analysis of ACE-Scan to longer (>12-mer) ACEs. Therefore, we used array densities greater than 10^{10} amines mm^{-2} for all ACE-Scan experiments reported here.

As a test of DA ligand specificity, ACE-Scan was also carried out on ATP DNA DAs subject to 10 mM GTP, a low-affinity purine ligand for the aptamer. No switching under 1 mM GTP, and limited switching under 10 mM GTP, was observed (**Supplementary Fig. 5**), which is consistent with past studies of ATP DNA DAs[e.g.1] and further supports our observation of ATP-specific induced fit ligand binding in this DA family.

Supplementary References

1 Li, N. & Ho, C.-M. Aptamer-based optical probes with separated molecular recognition and signal transduction modules. *J. Am. Chem. Soc.* **130**, 2380-2381 (2008).

2 SantaLucia, J. A unified view of polymer, dumbbell, and oligonucleotide DNA nearest-neighbor thermodynamics. *Proc. Natl. Acad. Sci. U.S.A.* **95**, 1460-1465 (1998).

3 Markham, N.R. & Zuker, M. DINAMelt web server for nucleic acid melting prediction. *Nucleic Acids Res.* **33**, W577-W581 (2005).

4 Schreck, J.S. *et al.* DNA hairpins destabilize duplexes primarily by promoting melting rather than by inhibiting hybridization. *Nucleic Acids Res.* **43**, 6181-6190 (2015).

5 Hekstra, D., Taussig, A.R., Magnasco, M. & Naef, F. Absolute mRNA concentrations from sequence‐specific calibration of oligonucleotide arrays. *Nucleic Acids Res.* **31**, 1962-1968 (2003).

6 Held, G.A., Grinstein, G. & Tu, Y. Modeling of DNA microarray data by using physical properties of hybridization. *Proc. Natl. Acad. Sci. U.S.A.* **100**, 7575-7580 (2003).

7 Munzar, J.D., Ng, A., Corrado, M. & Juncker, D. Complementary oligonucleotides regulate induced fit ligand binding in duplexed aptamers. *Chem. Sci.*, Advance Article (2017).

8 Russo Krauss, I. *et al.* High-resolution structures of two complexes between thrombin and thrombin-binding aptamer shed light on the role of cations in the aptamer inhibitory activity. *Nucleic Acids Res.* **40**, 8119-8128 (2012).
